# Multi-Omics Dissection of Drought Stress Responses in Crops: From Molecular Regulatory Networks to Climate-Resilient Breeding Applications

**DOI:** 10.3390/ijms27115008

**Published:** 2026-06-01

**Authors:** Baber Ali, Zeeshan Khan, Nijat Imin, Tibor Janda, Fatemeh Gholizadeh

**Affiliations:** 1 Faculty of Engineering, Computing and Science, Western Sydney University, Penrith, NSW 2751, Australia; b.ali@westernsydney.edu.au; 2 Research School of Biology, Australian National University, Acton, ACT 2601, Australia; 3 Atta-ur-Rahman School of Applied Biosciences (ASAB), National University of Sciences and Technology (NUST), Islamabad 44000, Pakistan; 4 Department of Plant Physiology and Metabolomics, Agricultural Institute, HUN-REN Centre for Agricultural Research, 2462 Martonvásár, Hungary; fatemeh.gholizadeh@atk.hun-ren.hu

**Keywords:** drought stress, multi-omics integration, genomics, transcriptomics, epigenomics, crop resilience, climate-smart agriculture, precision breeding

## Abstract

Drought stress is the most pervasive abiotic constraint on global crop productivity, with projected intensification under climate change threatening the yields of staple crops including wheat, rice, maize, and legumes. Conventional breeding approaches have delivered limited gains against drought tolerance, constrained by the polygenic and multifactorial nature of stress adaptation, the complexity of genotype-by-environment interactions, and the inadequacy of field-based phenotyping under variable stress conditions. Omics technologies, including genomics, transcriptomics, proteomics, metabolomics, epigenomics, and phenomics, have substantially advanced the molecular dissection of drought tolerance by enabling high-resolution characterization of stress-responsive genes, regulatory networks, adaptive proteins, and metabolic reprogramming pathways. Specific traits targeted include root system architecture and depth, osmotic adjustment capacity through proline and glycine betaine accumulation, antioxidant defense mechanisms, ABA-mediated stomatal regulation, LEA protein accumulation, epigenetic stress memory, and yield stability under water deficit. This review systematically examines omics-based strategies for drought stress mitigation across major crops, highlighting individual omics contributions, multi-omics integration frameworks, computational tools including machine learning and AI-driven predictive modelling, and translational breeding applications. Case studies in wheat, rice, maize, and legumes illustrate how omics-driven approaches accelerate precision breeding for drought resilience through marker-assisted selection, genomic selection, and CRISPR-based gene editing. Challenges including data integration complexity, high implementation costs, limited cross-species transferability, and the need for field-scale validation of microbiome-based strategies are critically addressed. Future perspectives encompassing single-cell and spatial omics, AI-driven predictive breeding, digital agriculture integration, and international data governance frameworks are discussed. By aligning with climate-smart agriculture principles, multi-omics approaches provide a robust and transformative foundation for developing drought-resilient crop cultivars suitable for water-limited production systems worldwide.

## 1. Introduction

Feeding a growing global population in the 21st century is increasingly challenged by the decline in arable land and freshwater availability, compounded by erratic weather patterns associated with climate change. Over recent decades, crop losses due to environmental extremes have progressively increased [1], with climate projections indicating greater frequency and intensity of droughts [2,3] and extreme heat events [1]. Modelling studies integrating agriculture with climate change scenarios project significant yield reductions in major staple crops, including wheat, rice, and maize, with severe consequences for global food production [4]. These projected losses are largely attributable to a complex of climate-related stresses, including heat waves, flooding, salinity, and water scarcity [5]. Among these, drought stress is the most widespread and devastating abiotic constraint on global agriculture, exerting long-term effects across large agro-ecological zones [6]. Water deficit impairs plant growth and productivity by inhibiting photosynthesis, nutrient assimilation, carbon metabolism, and reproductive development, with particularly irreversible damage occurring during sensitive developmental stages such as flowering and grain filling [7]. Drought impacts in the field are further compounded when plants are simultaneously exposed to biotic and additional abiotic stress. Furthermore, conventional crop production systems rely predominantly on water-intensive cultivation practices that are becoming increasingly unsustainable given accelerating urbanization, groundwater overexploitation, and progressive depletion of freshwater reserves. These challenges have created an urgent need to understand and improve drought tolerance in crops. However, conventional breeding and agronomic approaches have delivered limited gains, primarily because drought-responsive traits are polygenic and multifactorial. Drought adaptation is governed by numerous small-effect quantitative trait loci (QTLs), environment-sensitive gene expression patterns, and highly orchestrated physiological and molecular regulatory networks. Field phenotyping remains particularly constrained by environmental heterogeneity and temporal variation in stress intensity, limiting the effectiveness of selection [8,9]. Existing breeding methodologies are therefore insufficient to deliver the scale of improvement required under accelerating climate change, necessitating advanced molecular and biotechnological approaches.

**Figure 1:**
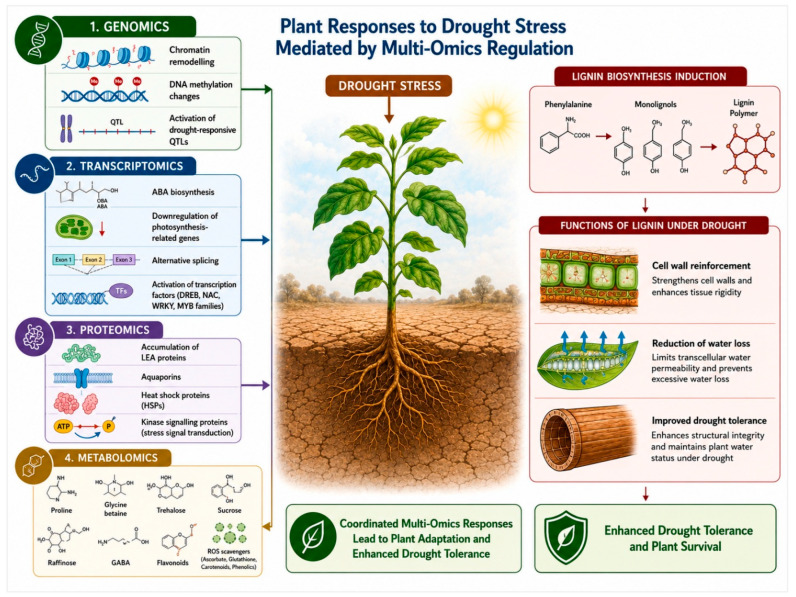
**Schematic illustration of plant responses to drought stress mediated by multi-omics regulation.** Drought stress induces systemic responses across four major omics layers. At the genomics level, responses include chromatin remodelling, DNA methylation changes, and activation of drought-responsive quantitative trait loci (QTLs). At the transcriptomics level, responses include abscisic acid (ABA) biosynthesis, downregulation of photosynthesis-related genes, alternative splicing, and activation of transcription factors including *DREB*, *NAC*, *WRKY*, and *MYB* families. At the proteomics level, responses include accumulation of late embryogenesis abundant (LEA) proteins, aquaporins, heat shock proteins (HSPs), and kinase signalling proteins involved in stress signal transduction. At the metabolomics level, drought stress triggers the accumulation of key protective metabolites including proline, glycine betaine, trehalose, sucrose, raffinose, gamma-aminobutyric acid (GABA), flavonoids, and reactive oxygen species (ROS) scavengers. Notably, lignin biosynthesis is also induced under drought stress, contributing to cell wall reinforcement, reduction of water loss through enhanced tissue rigidity, and improved drought tolerance by limiting transcellular water permeability. Together, these coordinated molecular responses across omics layers contribute to plant adaptation and tolerance under drought stress.

Omics technologies offer powerful tools for resolving the molecular basis of drought stress responses in plants (Figure 1). High-throughput platforms, including genomics, transcriptomics, proteomics, phenomics, and metabolomics, enable systematic identification of drought-responsive genes, regulatory factors, proteins, and metabolites involved in stress adaptation [10]. Critically, these technologies target specific and agriculturally relevant traits, including root system architecture and depth, osmotic adjustment capacity, antioxidant defense mechanisms, stomatal regulation and water-use efficiency, ABA-mediated signalling, LEA protein accumulation, and yield stability under water deficit [11,12]. These technologies have substantially advanced the molecular dissection of complex processes underlying drought tolerance, including osmotic adjustment, reactive oxygen species detoxification, hormonal regulation, and root system development. Moreover, integrative multi-omics methods offer system-level understanding of dynamic regulations of plant adaptation to water deficit [13]. These integrative approaches enable the identification of key regulatory hubs and biomarkers for breeding and genetic engineering of drought-resistant crops. The application of omics-based strategies in crop improvement programs therefore represents a powerful avenue for advancing multiple Sustainable Development Goals (SDGs), particularly the elimination of hunger under shifting climatic conditions.

Building on the capacity of high-throughput omics technologies to resolve drought-responsive regulatory networks, this review systematically examines omics-based strategies for improving drought tolerance in crops, elucidating the molecular, biochemical, and regulatory processes that govern stress adaptation. It further highlights integrative multi-omics frameworks and their translational applications in modern breeding, gene editing, and precision agriculture. Finally, the review addresses contemporary technical and analytical challenges and discusses future directions for harnessing systems biology and data-driven approaches to accelerate the development of climate-resilient crop cultivars. Unlike previous reviews, this work uniquely integrates single-cell and spatial omics perspectives, AI-driven predictive modelling frameworks, microbiome interactions, and precision agriculture strategies within a unified omics-to-breeding translational pipeline, providing a timely and comprehensive resource for advancing climate-resilient crop development.

## 2. Foundations of Omics Technologies in Drought Research

Omics technologies form the core platform of unravelling the multifaceted molecular structure of the responses of plants to drought stress [14]. Genomics, transcriptomics, proteomics, metabolomics and phenomics offer an integrative platform that allows high-resolution characterization of perceptions of stress, signal transduction and adaptive regulatory networks [15]. Such methods help in identifying stress-responsive genes, regulatory components, proteins and metabolic pathways which combine to regulate drought tolerance [11].

### 2.1. Genomics: Blueprint for Drought Tolerance

Genomics provides an effective roadmap to developing crops with improved tolerance to drought with elaboration of intricate genetic structures, the identification of adaptive alleles, and the accurate modification of traits [16]. It is a complex genetic method based on quantitative trait loci (QTL) mapping, genome-wide association studies (GWAS), and innovative technologies of genome editing, such as CRISPR/Cas, to produce climate-resistant crops. Quantitative trait loci mapping continues to be a pillar in the study of the genetic basis of drought-adaptive character like the root system structure and osmotic adjustment as a key predictor of water uptake and cellular homeostasis in drought conditions [17]. Significant QTL of root phenotypes such as total root length, dry weight and root tip number have been identified in wheat and rice which can account for large share of phenotypic variation during drought stress and hence used as markers in the breeding programs. For example, recent studies in wheat populations have successfully tagged QTL controlling drought-responsive root trait by using high density SNP arrays that allow the development of breeding-friendly markers to increase the drought resilient traits in elite lines [18]. Key QTLs identified through genomic studies across major crops are further summarized in Table 1. Likewise, root-length-linked and grain yield-linked multiple QTL have been discovered in rice concerning stress conditions, and these have offered valuable genomic loci to enhance drought resistance in the rain-fed ecosystem [19]. These QTLs may also interact with osmotic adjustment processes through osmolyte accumulation (discussed in Section 2.4), representing key genomic targets for drought-tolerant crop development.

Genome-wide association studies (GWAS) complement QTL mapping by screening natural and breeding populations across millions of genetic variants to identify loci associated with drought-response phenotypes. By exploiting historical recombination events across diverse germplasm, GWAS achieves finer mapping resolution and captures broader allelic diversity compared with biparental QTL mapping approaches [20]. In rice, GWAS conducted under osmotic stress conditions has identified multiple significant loci regulating root traits, including primary root length and root surface area, several of which encode previously uncharacterized genes, underscoring the utility of GWAS in detecting environmentally responsive alleles [21]. Notably, specific genes and QTLs associated with drought tolerance have been identified through GWAS in several major crops. In rice, the gene *OsDREB1C*, identified through haplotype-based GWAS, confers improved drought tolerance and grain yield under stress conditions [22]. Deep rooting gene *DRO1* has also been associated with drought avoidance through QTL-based mapping and validated functionally in rice, demonstrating its role in directing root growth angle and enhancing water uptake from deeper soil layers [23]. In maize, GWAS studies have identified SNP markers and candidate genes associated with root cortical aerenchyma formation and stomatal conductance, both of which contribute to water-use efficiency under drought [24]. In wheat, GWAS has identified multiple SNP markers and candidate genes associated with root morphology, canopy temperature, and leaf rolling under water stress, including the transcription factor gene *TaMYB7-A1* linked to root depth and drought resistance, providing validated genomic selection targets [25,26]. In soybean, QTL mapping studies have revealed loci associated with osmotic adjustment, proline accumulation, and ABA-responsive gene expression under drought, with candidate genes including those encoding LEA proteins and protein kinases involved in stress signalling [27]. Collectively, the allelic diversity captured by GWAS provides a genomic catalogue of natural variation that plant breeders can exploit through marker-assisted selection or gene editing to assemble drought-adaptive trait combinations in elite cultivars.

Emerging genome editing technologies like CRISPR/Cas have transformed the way we use that knowledge with exact and targeted editing of the genes that control stress responses. CRISPR/Cas systems (CRISPR/Cas9, base editing and prime editing) enable researchers to knock out negative regulators, turn on positive alleles or precisely tune gene expression without introducing any foreign DNA, which will expedite the development of drought tolerant cultivars [28]. Experimental studies have demonstrated that CRISPR-based editing of key drought-stress regulatory genes can substantially improve drought tolerance across multiple crop species. In rice, CRISPR-Cas9-mediated knockout of the *OsDST* gene, a negative regulator of drought tolerance, reduced stomatal density and enhanced leaf water retention under water deficit conditions [29]. CRISPR-Cas9-mediated knockout of *OsERA1*, a negative regulator of ABA signalling, enhanced ABA sensitivity and promoted stomatal closure under drought stress conditions, resulting in improved drought tolerance without compromising leaf growth in rice [30]. For example, drought sensitive regulatory genes in maize have been edited through CRISPR/Cas9 leading to lines with better root development, reduced water loss and enhanced antioxidant defense as well as yield stability under drought conditions, which demonstrates the power of genome editing in converting genomic knowledge to agronomic performance [31]. Similarly, the gene editing of *GmHdz4* gene using Cas9 and its corresponding guide RNA showed a significant positive effect on the drought tolerance of soybean by optimizing root system architecture, accumulation of osmolytes and antioxidant defence mechanism [32]. It further highlights progress in the technologies for using the CRISPR technique, as well as combining these with high-throughput phenotyping and genomic selection to enable faster climate-resilient crop breeding.

### 2.2. Transcriptomics: Gene Expression Under Water Deficit

Transcriptomics, the study of genome-wide RNA expression, provides detailed information about the regulation of gene expression under drought stress conditions and identifies important molecular pathways that allow plants to acclimate to drought stress and survive in water-limited environments [33,34]. Transcriptional profiling of stress responses can reveal genes and regulatory factors and interaction pathways resistant to stress, which initiate adaptive response, providing the basis of targeted genetic enhancement of crop resilience. One of the pioneering works in this field was demonstrating the overlapping mechanisms between the main abiotic stressors, drought, cold and high salinity, using the microarray technique [35].

RNA sequencing (RNA-seq) is now the principal instrument of plant drought transcriptomics as it is the most sensitive, accurate and able to provide the whole transcriptome when no prior sequence data are available. In the drought condition, RNA-seq analysis reveals differentially expressed genes (DEGs), hormone-signalling genes, osmotic-adjusting genes, antioxidant-defence genes, as well as cell-protective genes [36]. Among hormonal regulators, abscisic acid (ABA) is the primary stress hormone mediating drought responses through a well-characterized signalling cascade involving *PYR/PYL/RCAR* receptors, PP2C phosphatases, and SnRK2 kinases, which collectively regulate stomatal closure, osmotic adjustment gene expression, and root growth adaptation under water deficit [37]. Transcriptome profiling during drought in maize showed that thousands of DEGs were observed between tolerant and sensitive genotypes, suggesting massive reorganization of the transcriptome in relation to survival mechanisms induced by stress such as photosynthetic maintenance and metabolic regulation [38]. Transcriptomic studies of alfalfa cultivars have suggested that improvement of antioxidant activity and control of major metabolic processes are important factors in drought tolerance [39]. Although analysis of specific DEGs can give useful information, it is necessary to get insights into the interaction between diverse DEGs to understand more complex drought-related regulatory processes. Gene co-expression network analysis allows identifying functional related modules of genes through clustering the genes that have similar patterns of expression under various set of conditions in which the genes are the nodes and their expression relationship with the edges. Such method will help to identify the presence of the coordinated regulatory pathway and important hub genes of the responses to drought stress [40]. A network analysis such as the Weighted Gene Co-expression Network Analysis (WGCNA) of transcriptome data can find slated network modules that are significantly correlated with drought tolerance phenotypes [41]. These modules frequently include hub genes (very connected regulators) including *NAC*, *WRKY* and *AP2/ERF* transcription factors that have critical roles in stress response pathways.

Transcription factors (TFs) are proteins that bind sequences of DNA to control gene expression to serve as global master switches in coping with stress. Some of the best-known TF families investigated in research on drought include the DREB (Dehydration-Responsive Element Binding) and NAC (NAM, ATAF, and CUC) families [42]. Combining transcriptome information with functional analyses has also shown that DREB TFs play a major role in mediating ABA-independent stress responses, and *NAC* TFs mediate abiotic stresses signalling and developmental mechanisms, which also include root growth, leaf senescence, and cell wall change [43]. Their discovery along with other TF families (e.g., WRKY, MYB) by RNA-seq makes clear the significance of transcriptional regulation in drought resistance and validates the potential to use the targeted regulators, which can be improved genetically or through biotechnology. A summary of key transcriptomic signatures, transcription factors, and quantitative trait loci associated with drought tolerance across major crops is provided in Table 1.

**Table 1:** Key transcriptomic signatures, transcription factors, and quantitative trait loci associated with drought tolerance in major crops.

Crop	Omics Layer	Gene/TF/QTL	Function in Drought Tolerance	Reference
Rice	Transcriptomics	*LEA4-5*, *LEA14*	Late embryogenesis abundant proteins; cellular protection during dehydration	[44]
Rice	Transcriptomics	*OsRab16A*	Stress-responsive gene; ABA-dependent drought signalling	[44]
Rice	Transcriptomics	*OsADF3*	Actin depolymerizing factor; cytoskeletal reorganization under stress	[44]
Rice	Transcriptomics	*OsPP108*	Protein phosphatase; ABA signalling regulation	[44]
Rice	Transcriptomics	*GBF3*	G-box binding factor; ABA-dependent transcriptional regulation	[44]
Rice	TF	*OsDREB1C*	DREB transcription factor; ABA-independent stress response, improved grain yield under drought	[22]
Rice	QTL	*DRO1*	Deep rooting QTL; controls root growth angle and enhances water uptake from deeper soil layers	[23]
Rice	QTL	Root length and surface area QTLs	Root morphology traits; osmotic adjustment and water uptake under drought	[21]
Wheat	Transcriptomics	DREB family genes	Dehydration-responsive element binding; ABA-independent osmotic stress signalling	[43]
Wheat	Transcriptomics	NAC family genes	Abiotic stress signalling; root growth, leaf senescence, and cell wall modification	[43]
Wheat	TF	*TaMYB7-A1*	MYB transcription factor; root depth enhancement and drought resistance	[26]
Wheat	QTL	Root morphology QTLs	Root length, dry weight, and tip number; phenotypic variation under drought stress	[18]
Wheat	QTL	SNP-tagged drought QTLs	Water-use efficiency and canopy temperature regulation under water stress	[25]
Maize	Transcriptomics	Photosynthesis and metabolic genes	Transcriptome reorganization; maintenance of photosynthesis and metabolic regulation under stress	[38]
Maize	TF	WRKY and AP2/ERF families	Stress-responsive transcriptional regulation; ROS detoxification and osmotic adjustment	[42]
Soybean	Transcriptomics	*FT1*, *CCR1L*, *RPL18* isoforms	Alternative splicing variants; post-transcriptional regulation of drought adaptation	[45]
Soybean	TF	*GmHdz4*	HD-Zip transcription factor; root system architecture, osmolyte accumulation, antioxidant defense	[32]
Alfalfa	Transcriptomics	Antioxidant and metabolic genes	Enhanced antioxidant activity and metabolic regulation contributing to drought tolerance	[39]
Rapeseed	TF	*MYB*, *CER1*, *FAR3*, *MAH1*	Cuticular wax biosynthesis; lipid metabolism and hormone signalling linked to drought tolerance	[46]
Cotton	TF	*HSF*, *bHLH*, *C2H2*, *B3*, *Tify*, *AUX/IAA*, *ARR-B*	Regulatory network controlling genotype-specific drought response	[47]

### 2.3. Proteomics: Functional Machinery Under Stress

Proteomics can offer a complete picture of the functional protein machinery underlying responses of plants to stress, including not only a change in protein abundance, but also the dynamics of protein activity and interaction networks likely to be central to stress adaptation [48]. Proteomic analyses have repeatedly reported an improved expression of antioxidant enzymes like superoxide dismutase (SOD), catalase (CAT), peroxidases, and glutathione-S-transferases (GST) that are important in the detoxification of ROS and protect cellular integrity in the case of water deficit [49]. Proteomics also shows the up regulation of molecular chaperones, such as heat shock proteins (HSPs) which help in the proper folding of the protein, preventing aggregation and stabilizing the proteome in stress conditions [50,51]. These stress-responsive proteins play a key role in metabolic homeostasis during the process of dehydration and rehydration to enable the further growth of plants in unfavorable conditions.

Proteomics does not only determine the abundance of proteins but also identifies protein post-translational modification (PTM) including phosphorylation, ubiquitination and acetylation through which protein activity, stability and signalling are critical to drought stress [52]. PTMs can modulate enzyme activities, switch protein localization or label proteins to be degraded which allows them to fine-tune their functional roles in stress responses. Indicatively, phosphorylation processes tend to regulate the functions of signalling proteins, transcription factors and enzymes involved in metabolism, which play a major role in adaptation to drought [53]. PTM-based proteomic studies enable researchers to track dynamic changes in protein modification states and provide mechanistic studies of how plants adapt rapidly to water deficit by modifying their proteome. These adjustments are a quick stratum of control, which makes plants respond effectively to the changing environmental conditions [53]. Moreover, proteomic studies also help to build protein-protein interaction (PPI) networks to incorporate individual proteins into wider regulatory models of drought response. PPI networks are characterized by the existence of nodes (proteins) and the existence of edges (physical or functional interactions among proteins) that allow identifying hub proteins as main central regulators of stress signalling pathways [54,55]. Drought-specific PPI research on crop species such as rice has demonstrated both interaction networks among transcription factors, signalling proteins and stress regulators which orchestrate to maintain protective and adaptive mechanisms in the presence of water deficit conditions. These networks highlight how the proteins in the cell interact to activate complex responses as well as pinpointing regulatory modules important in the development of drought tolerance [54].

### 2.4. Metabolomics: Chemical Fingerprints of Drought Response

Metabolomics, or the global analysis of small molecules, or metabolites, in biological systems is a way of getting a direct readout of the biochemical adjustments that plants make in response to drought stress [56]. By profiling metabolite changes, researchers can identify some of the major adaptive strategies such as build-up of osmolytes, small organic molecules that aid the cellular water balance and macromolecule protection against dehydration. Key osmolytes including proline, glycine betaine, trehalose, and sucrose accumulate during drought stress and contribute to osmotic adjustment, ROS scavenging, and cellular protection. In addition to these osmolytes, lignin biosynthesis is induced under drought stress as part of the cell wall remodelling response, contributing to reduced water loss through enhanced tissue rigidity and limited transcellular water permeability, thereby playing an important role in drought tolerance [57]. Metabolomic studies have consistently confirmed elevated levels of osmolytes particularly proline and glycine betaine in drought-tolerant compared with drought-sensitive genotypes, validating their utility as biochemical biomarkers of drought adaptation given their well-established roles in osmotic adjustment and cellular protection [58]. For example, targeted and untargeted metabolite profiling of crops, such as wheat [59], rice [60], and maize [61] proved the up regulation of osmolytes in an integrated drought stress response. Metabolites that show a correlation with stress tolerance phenotypes are identified as biomarkers. Biomarkers can yield quick and cheap screening methods for breeders to select drought tolerant genotypes without long field trial. For example, an increase in various metabolites, like proline, raffinose, γ-aminobutyric acid (GABA) and some flavonoids have been linked to increased drought tolerance in several plant species [62].

In addition to the accumulation of osmolytes, drought stress provokes massive metabolic reprogramming to save energy and redirect metabolic fluxes into tolerance to stress [63]. At water deficit, most plants will tend to hinder growth related pathways and promote pathways connected with energy conservation, and stress defense, including changed carbohydrate metabolism, improved lipid metabolism, and increased secondary metabolites. Metabolomic studies have shown a decreased central carbon metabolism intermediate (e.g., glycolysis and the tricarboxylic acid cycle) and an increased metabolic state of stress signalling and production of antioxidants [63]. These shifts are helpful to plants to balance energy demand with limited energy resources, maintain cellular redox state and survival rather than existence and growth. Modification of the metabolic processes may also significantly contribute to increased crop production. The process of photorespiration, which results from oxygenation of RuBP by RuBisCO, may become more prevalent at higher temperatures or under drought conditions. It has been shown in tobacco plants that creating more efficient photorespiratory pathways via inhibiting the native pathway may substantially increase both photosynthetic efficiency and biomass production [64]. This experiment can serve as a model for increasing the yield of other economically important C3 crops. Recent metabolomic studies in drought stressed rice [65], barley [66], soybean [67] have also shown down-regulation of growth-promoting metabolites and up-regulation of stress-associated metabolites, indicating a strategic reallocation of biochemical resources.

### 2.5. Epigenomics: Regulatory Layer Beyond DNA Sequence

Epigenomics has emerged as a critical regulatory layer in plant drought responses, governing gene expression through reversible and heritable chemical modifications of DNA and chromatin that operate independently of changes in the underlying DNA sequence [68]. These epigenetic mechanisms enable plants to fine-tune transcriptional programs in response to water deficit with a precision and reversibility that transcriptional regulation alone cannot achieve, and they underpin the phenomenon of stress priming, whereby prior drought exposure confers enhanced tolerance upon subsequent stress encounters.

#### 2.5.1. DNA Methylation and Stress Memory

DNA methylation, primarily occurring at cytosine residues in CG, CHG, and CHH sequence contexts, is one of the most extensively studied epigenetic modifications in drought-stressed plants. Drought-induced changes in cytosine methylation patterns at gene promoters, gene bodies, and transposable elements modulate transcriptional activity of stress-responsive loci. Genome-wide bisulfite sequencing studies in maize have demonstrated that drought stress induces locus-specific hypermethylation and hypomethylation events, with locus-specific hypomethylation at promoters of stress-responsive genes correlating with their transcriptional activation under water deficit [69]. In rice, genome-wide bisulfite sequencing has revealed widespread drought-induced DNA methylation changes at gene promoters and transposable elements in contrasting tolerant and sensitive genotypes, with differentially methylated regions associated with stress-responsive genes implicated in osmotic adjustment and stomatal regulation [70]. Critically, some of these methylation changes persist after stress relief, establishing an epigenetic stress memory that primes faster and stronger transcriptional responses upon repeated drought exposure [68,71]. This memory is mediated in part through the maintenance methyltransferase MET1 and the RNA-directed DNA methylation (RdDM) pathway involving DOMAINS REARRANGED METHYLTRANSFERASE 2 (DRM2), which together preserve stress-induced methylation states across cell divisions. Methylation-sensitive amplification polymorphism (MSAP) analysis has been widely applied as a cost-effective technique for genome-wide assessment of cytosine methylation patterns in crop species under drought stress, complementing whole-genome bisulfite sequencing approaches by enabling rapid screening of differentially methylated loci across large germplasm [72,73].

#### 2.5.2. Histone Modifications and Chromatin Remodelling

Histone modifications represent a second major epigenetic regulatory mechanism controlling drought-responsive gene expression. Post-translational modifications of histone proteins, including acetylation, methylation, phosphorylation, and ubiquitination, alter nucleosome configuration and chromatin accessibility, directly influencing the transcriptional competency of target genes. Active histone marks, particularly histone H3 lysine 4 trimethylation (H3K4me3) and histone H3 lysine 9 acetylation (H3K9ac), are associated with open chromatin states and transcriptional activation of drought-responsive genes. In *Arabidopsis*, H3K4me3 enrichment at the promoters of drought-inducible genes including *RD29A*, *RD29B*, and *RAB18* has been shown to facilitate their rapid transcriptional activation upon water deficit, with this enrichment maintained as part of stress memory [74]. Conversely, the repressive mark H3K27me3, deposited by Polycomb Repressive Complex 2 (PRC2), maintains transcriptional silencing of growth-related genes during drought-induced metabolic reorganization, allowing reallocation of cellular resources toward stress tolerance mechanisms. Histone deacetylases (HDACs), such as HDA6 and HDA19 in *Arabidopsis*, and their crop orthologs actively modulate histone acetylation states under drought, with loss-of-function mutants displaying altered stress-responsive gene expression and compromised drought tolerance. In *Arabidopsis*, chromatin remodelling complexes involving the SWI/SNF ATPase BRAHMA (BRM) regulate ABA responsiveness by modulating chromatin accessibility at ABA-responsive element (ABRE)-containing promoters, highlighting the direct mechanistic link between chromatin remodelling and drought adaptation [75].

#### 2.5.3. Small RNA-Mediated Epigenomic Regulation

Small RNAs, including microRNAs (miRNAs) and small interfering RNAs (siRNAs), constitute a third epigenomic regulatory layer that fine-tunes gene expression post-transcriptionally and contributes to RdDM-mediated transcriptional silencing during drought stress. Biogenesis of drought-responsive miRNAs involves the coordinated activity of RNA-DEPENDENT RNA POLYMERASE (RDR), DICER-LIKE (DCL), and ARGONAUTE (AGO) proteins, which have been shown to be transcriptionally activated in the early phases of drought stress in maize [76]. Among the best-characterized drought-responsive miRNAs, miR169 targets *NUCLEAR FACTOR Y* subunit A (*NFYA*) transcription factors, and its downregulation under drought stress in Arabidopsis and rice releases *NFYA*-mediated activation of drought-responsive genes including *NCED3*, a key enzyme in ABA biosynthesis. miR396 targets *GROWTH-REGULATING FACTOR* (*GRF*) transcription factors and its drought-induced upregulation in rice reduces cell proliferation while redirecting metabolic resources toward stress tolerance. miR159 targets MYB transcription factors including *MYB33* and *MYB65* in *Arabidopsis*, modulating ABA sensitivity and germination responses under water deficit. In wheat, miR408 is among the drought-responsive miRNAs that target copper-related genes including plastocyanin-like transcripts, with its expression significantly altered under water deficit conditions [77]. Meanwhile, siRNAs participate in RdDM-mediated transcriptional silencing of transposable elements activated by drought stress, maintaining genome stability and preventing aberrant transposon-driven transcriptional noise during water deficit [78]. High-throughput small RNA sequencing has identified hundreds of drought-regulated miRNAs and siRNAs across rice, wheat, maize, and soybean, underscoring the pervasive role of small RNA-mediated epigenomic regulation in coordinating environmental cues with gene regulatory networks.

#### 2.5.4. Epitranscriptomics: RNA Modifications Under Drought

An emerging dimension of epigenomic regulation in drought stress involves chemical modifications of RNA molecules, collectively termed the epitranscriptome. N6-methyladenosine (m6A) is the most abundant internal modification of messenger RNA and has been recently identified as a regulator of mRNA stability, splicing, and translation efficiency under abiotic stress conditions. In *Arabidopsis*, the m6A machinery including writers (MTA, MTB), erasers (ALKBH), and readers (ECT2, ECT3) has been characterized and shown to regulate mRNA stability and translational output [79]. Emerging evidence suggests that m6A modification of stress-responsive transcripts including those involved in ABA signalling contributes to drought stress responses, though the full scope of the drought-responsive epitranscriptome remains under active investigation [80].

### 2.6. Phenomics: Linking Molecular Insights to Traits

Phenomics bridges the gap between molecular-level omics data and observable drought-adaptive traits by enabling high-resolution, high-throughput quantification of complex physiological and morphological responses at the whole-plant and canopy scales [81]. Given that drought tolerance is ultimately expressed as a measurable phenotype under field conditions, phenomics represents a critical bottleneck in translating molecular discoveries into breeding outcomes, and its integration with other omics layers is essential for building robust genotype-to-phenotype predictive models.

#### 2.6.1. Aerial and Canopy Phenotyping Platforms

Unmanned aerial vehicle (UAV)-based remote sensing platforms equipped with thermal infrared, hyperspectral, RGB, and multispectral cameras have transformed large-scale field phenotyping of drought responses by enabling non-destructive, high-throughput measurement of canopy-level traits across hundreds of genotypes simultaneously [82]. Thermal infrared imaging captures canopy temperature, a reliable surrogate for stomatal conductance and transpiration rate, with drought-tolerant genotypes typically maintaining cooler canopies through sustained stomatal function under water deficit. Hyperspectral imaging measures leaf reflectance across hundreds of wavelengths, enabling the derivation of vegetation indices sensitive to chlorophyll content, water status, and photosynthetic efficiency, including the normalized vegetation index (NDVI), photochemical reflectance index (PRI), and water band index (WBI). Chlorophyll-a fluorescence imaging, including pulse amplitude modulated (PAM) fluorimetry, provides non-invasive measurements of photosystem II efficiency (Fv/Fm) and non-photochemical quenching, which are sensitive indicators of drought-induced photoinhibition and oxidative stress. Recent UAV-based phenotyping studies in wheat and maize have demonstrated that high-throughput canopy temperature and NDVI measurements collected during the reproductive stage are significantly correlated with grain yield under drought, validating their utility as selection criteria in breeding programs [83,84].

#### 2.6.2. Root Phenotyping Platforms

Root system architecture is a primary determinant of drought avoidance through deeper water extraction, yet roots remain challenging to phenotype due to their inaccessibility in soil. X-ray computed tomography (CT) scanning enables three-dimensional reconstruction of root systems in intact soil columns, providing detailed measurements of root depth, branching angle, root length density, and root tip number without destructive sampling [85]. Rhizotron systems and shovelomics approaches facilitate high-throughput assessment of root architectural traits in field conditions, with recent applications in maize and sorghum identifying genotypic variation in root cortical aerenchyma formation and steep root angle as key contributors to drought avoidance. Ground-penetrating radar is emerging as a promising non-invasive tool for field-scale root phenotyping, offering the potential to estimate rooting depth and biomass distribution across large populations. These root phenotyping platforms have revealed that deep rooting angle, high root length density in subsoil layers, and reduced root cortical burden are consistently associated with improved water extraction and yield stability under drought across multiple crop species [85,86].

#### 2.6.3. Integration of Phenomics with Multi-Omics Data

The true power of phenomics in drought research is realized when phenotypic datasets are integrated with genomic, transcriptomic, and metabolomic data to construct predictive models linking molecular variation to field performance. Genome-wide association studies combining high-density SNP arrays with UAV-derived phenomic traits have identified genomic loci associated with canopy temperature depression, stay-green, and water-use efficiency in wheat and sorghum, providing validated molecular markers for drought-adaptive phenotypes [11,87]. Machine learning models trained on combined SNP marker profiles and UAV-derived phenomic measurements have significantly improved the accuracy of genomic selection for drought-adaptive traits in maize and wheat compared with models based on genomic data alone [11,87]. Transcriptome-phenome integration studies have further identified regulatory modules linking the expression of drought-responsive transcription factors including DREB and NAC family members with specific physiological phenotypes including stomatal conductance, relative water content, and root depth, providing mechanistic links between molecular regulation and adaptive performance. Additionally, metabolome-phenome correlations have identified proline accumulation, soluble sugar content, and antioxidant enzyme activity as robust biochemical markers that predict osmotic adjustment capacity and yield stability under drought, enabling early-stage biochemical screening of breeding populations. The continued development of high-throughput, low-cost phenotyping platforms and their systematic integration with multi-omics datasets through advanced computational frameworks represents one of the most promising avenues for accelerating drought-resilient crop breeding.

## 3. Multi-Omics Integration for Drought Stress

**Figure 2:**
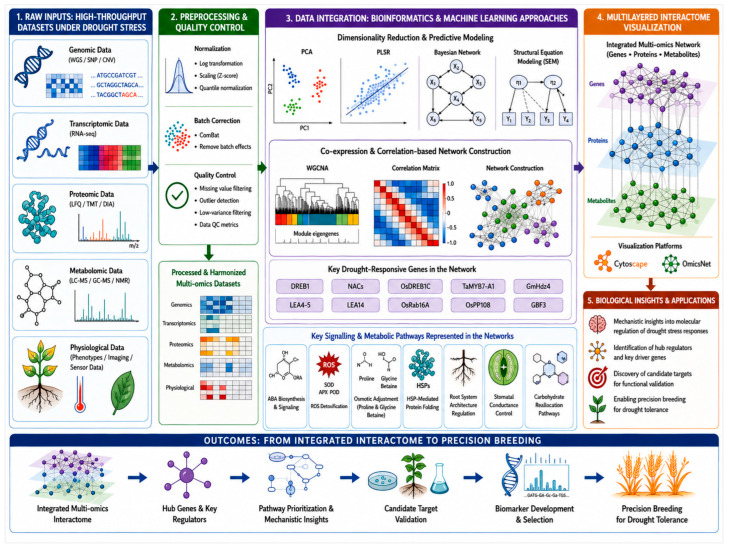
**Multi-omics data integration workflow for drought stress analysis.** High-throughput datasets, including genomic, transcriptomic, proteomic, metabolomic, and physiological data, are acquired as raw inputs and subjected to preprocessing steps including normalization, batch correction, and quality control. The processed datasets are then integrated using bioinformatics and machine learning approaches, including dimensionality reduction techniques such as principal component analysis (PCA) and partial least squares regression (PLSR), as well as Bayesian network modelling and structural equation modelling. Co-expression and correlation-based methods, including weighted gene co-expression network analysis (WGCNA), are applied to construct interaction networks. Key drought-responsive genes incorporated in these networks include *DREB1*, *NAC* transcription factors, *OsDREB1C*, *TaMYB7-A1*, *GmHdz4*, *LEA4-5*, *LEA14*, *OsRab16A*, *OsPP108*, and *GBF3*. Key signalling and metabolic pathways represented in the networks include ABA biosynthesis and signalling, reactive oxygen species (ROS) detoxification, osmotic adjustment through proline and glycine betaine accumulation, heat shock protein (HSP) mediated protein folding, root system architecture regulation, stomatal conductance control, and carbohydrate reallocation pathways. The resulting interactome is visualized as multilayered regulatory networks comprising genes, proteins, and metabolites using platforms such as Cytoscape 3.10.4 and OmicsNet 2.0, providing mechanistic insights into the molecular regulation of drought stress responses and enabling identification of hub regulators and candidate targets for precision breeding.

Multi-omics integration has become a new paradigm of explaining the intricate regulatory networks of plants response to drought stress by integrating genomics, transcriptomics, proteomics, metabolomics, and phenomics into a single paradigm of analysis as depicted in Figure 2. Current improvements in high-throughput sequencing, artificial intelligence, and machine learning have enabled integration of multi-dimensional data to improve predictive quality in characterizing drought-resilient genotypes and rapid emphasis on precision breeding plans.

### 3.1. Systems Biology Framework

Systems biology provides a global model of plant drought stress by combining molecular information on several biological levels and using more complex computational methods to identify emergent characteristics of complex stress responses [88]. Multi-layered interaction networks integrating gene expression, protein abundance, and metabolite profiles have helped to identify core regulatory hubs and modules that coordinate cellular responses to water deficit. For example, in maize, multi-omics integration has identified large-scale drought resistance gene networks and key regulatory factors coordinating stress responses [89,90]. Similarly, in rice, multi-omics integration combining genomics, transcriptomics, proteomics, and metabolomics has revealed coordinated regulatory networks governing drought tolerance, including ABA-mediated signalling hubs and osmotic adjustment pathways, providing a system-level understanding of rice drought adaptation [89,90]. In this context, mechanistic mapping of key stress-adaptive pathways, including ABA-mediated signalling (described in Section 2.2), ROS detoxification (described in Section 2.3), and carbohydrate reallocation, can be performed through pathway reconstruction using integrated omics data and curated biochemical databases [91]. Dynamic models, such as constraint-based metabolic modelling, ordinary differential equation (ODE) systems, and machine learning-driven simulations can predict the responses and flux variations of water deficit over time and discover the non-linear dynamics of the regulatory networks and can predict the genotype performance in drought [92,93]. These biology methods not only enhance our current basic knowledge of the molecular structure of drought tolerance but also facilitate translational practice by informing the rational design of breeding objectives and biotechnological interventions to help crop species to become more resilient.

### 3.2. Computational Tools and Pipelines

Machine learning algorithms, including random forests, support vector machines, deep neural networks, and ensemble models, are increasingly applied to integrate genomics, transcriptomics, proteomics, metabolomics, and phenomics data for predicting drought-adaptive traits, detecting biomarkers, and classifying genotypes by stress resilience. In a concrete application, Chen et al. [94] applied GWAS combined with machine learning to identify SNP markers and candidate genes associated with drought tolerance at the seedling stage in a maize association panel of 379 inbred lines under field water-deficit conditions, demonstrating how integrating marker data with field phenotypic data improves genomic prediction accuracy beyond traditional statistical approaches. Similarly, a transcriptome-based machine learning study in maize used random forest models trained on RNA-seq expression profiles to predict complex drought-adaptive traits, identifying key regulatory transcription factors as primary predictive features and validating their functional importance through network analysis [95]. Robust data harmonization and normalization are prerequisites for reliable machine learning performance in multi-omics integration. Methods including quantile normalization, variance stabilization, standardization, and empirical Bayes correction such as ComBat ensure that multi-omics features are comparable and biologically interpretable across heterogeneous datasets, which is indispensable for downstream integration and predictive modelling [96]. Without proper harmonization, spurious correlations and model overfitting can obscure genuine biological signals, particularly when integrating datasets generated by different technologies or experimental designs. For example, metabolomic profiling of drought-tolerant and drought-sensitive wheat genotype seedlings revealed that proline biosynthesis and phenylpropanoid pathways are key metabolic response modules under water deficit, with significant metabolite differences between tolerant and sensitive genotypes demonstrating that data normalization is critical for accurate biological interpretation [97]. After data has been normalized, integrative visualization and network analysis software like Cytoscape 3.10.4 and OmicsNet 2.0 can be used to offer strong platforms to prevail and comprehend multi-layered biological networks in drought stress. In practice, Cytoscape 3.10.4 has been used to construct and visualize gene-protein-metabolite interaction networks in drought-stressed crops by importing co-expression data from WGCNA analyses, superimposing differential expression values as node attributes, and applying the CytoHubba plugin to rank hub genes by connectivity centrality. This workflow identified *NAC* and *WRKY* transcription factors as primary hub regulators of drought response modules in rice and maize, with their downstream targets enriched in ABA signalling and ROS detoxification pathways [98,99]. OmicsNet 2.0 extends this approach by enabling simultaneous integration of transcriptomic, proteomic, and metabolomic layers into a unified 3D interactive network, with its application to plant stress datasets enabling cross-omics visualization of signalling cascades and metabolic pathways that would not be apparent from single-omics analyses alone [100]. Such platforms do not only increase interpretability but also allow generation of hypotheses and prioritize candidate genes and pathways to validate these hypotheses.

Despite their considerable promise, machine learning approaches in multi-omics drought research are subject to several important limitations that must be critically acknowledged. First, overfitting represents a fundamental risk when high-dimensional multi-omics datasets containing thousands of molecular features are used to train models on relatively small sample sizes, which is common in drought research where generating large phenotyped populations under controlled stress conditions is logistically and financially demanding. Overfitted models perform well on training data but fail to generalize to independent populations or environments, severely limiting their practical utility in breeding programs [95]. Rigorous cross-validation strategies, including k-fold cross-validation, leave-one-out cross-validation, and independent test set validation, are therefore essential components of any machine learning pipeline applied to drought omics data. Second, the biological interpretability of complex machine learning models, particularly deep neural networks and ensemble methods, remains a significant challenge. These models function as black boxes in which the relationship between input molecular features and predicted phenotypic outcomes is not mechanistically transparent, making it difficult to extract biologically meaningful insights or generate testable hypotheses about drought tolerance mechanisms [101]. Feature importance scores and explainability frameworks such as SHapley Additive exPlanations (SHAP) and Local Interpretable Model-agnostic Explanations (LIME) are increasingly being applied to improve the biological interpretability of machine learning outputs in plant omics research, enabling researchers to identify which molecular features most strongly drive model predictions and linking these to known stress response pathways [102]. Third, independent validation of machine learning predictions in diverse genetic backgrounds and field environments remains insufficiently practiced in drought omics research. Most published models are validated within the same dataset or population used for training, limiting confidence in their transferability across germplasm, environments, and cropping systems. Future machine learning studies in drought research should prioritize multi-environment, multi-population validation to demonstrate the robustness and generalizability of predictive models before their deployment in breeding decision support systems [103].

### 3.3. Statistical Approaches

Statistical methods play a central role in deriving biologically significant trends in high-dimensional drought-related multi-omics data to simplify the analysis, combine various data sources, and identify the regulative mechanisms of stress responses. Dimensionality reduction techniques, namely Principal Component Analysis (PCA) and Partial Least Squares Regression (PLSR) are currently popular dimension reduction methods of converting huge data to a collection of orthogonal elements that capture the largest volume of variance to visualize, categorize, and associate traits. For example, PCA has been used for the transcriptomic and metabolomic profiles of drought stressed wheat to discriminate tolerant and sensitive genotypes and to identify the important variables controlling stress phenotypes [104]. While, PLSR has been applied to relate spectral phenomics data to physiological drought indicators including relative water content and yield parameters in maize [105]. In addition to the use of linear projection, Bayesian inference frameworks have been effectively used to combine multi-omics data in a probabilistic manner, including prior biological knowledge and uncertainty modelling to predict latent structure and better predictive performance of drought adaptation phenotypes. A concrete example of successful multi-omics integration using these statistical frameworks was reported in drought-stressed soybean, where combined transcriptomic and metabolomic profiling identified tryptophan, proline, and flavonoid biosynthesis as key drought-responsive pathways, with amino acid metabolism and phenylpropanoid biosynthesis genes emerging as central regulatory nodes coordinating transcriptional and metabolic drought responses [106]. Bayesian network models have been effectively used to predict gene-regulatory relationships under abiotic stress to model probabilistic relationships between transcription factors, metabolites, and phenotypes [107]. Causal modelling approaches, including structural equation modelling (SEM) and causal graphical modelling, enable separation of direct and indirect regulatory effects, identifying hubs that causally drive drought response outcomes rather than merely correlating with them. Causal inferences on integrated omics data used on rice and *Arabidopsis* have identified important regulators like ABA signalling components and ROS detoxification mediators as primary causal regulators of drought tolerance, consistent with findings described in Section 2.2 and Section 2.5 [108]. These statistical frameworks collectively enhance the interpretability of multi-omics data through dimensionality reduction, probabilistic modelling, and causal inference, providing a quantitative basis for identifying mechanistic drivers of stress resilience and prioritizing targets for functional validation in drought-affected agricultural systems.

### 3.4. Visualization and Interpretation

More sophisticated visualization and interpretation methods, including heatmaps, network graphs, interactive dashboards, etc., are now essential to interpret complex multi-omics data related to drought stress, and to integrate different omics tools into coherent biological findings (Figure 2). Common techniques used to determine patterns of differential expression and stress-respective molecular signatures include heatmaps and hierarchical clustering analysis which are frequently used to find significant discriminant compounds, including phenylpropanoids, amino acids, and hormones, in response to stress [109]. Integrative systems biology methods have been used in practice to construct network graphs identifying gene regulatory networks, protein-protein interactions, and metabolite-pathway relationships in drought-stressed crops. For example, Integrative systems biology methods combining transcriptomics, proteomics, and metabolomics have been applied in drought-stressed crops to construct gene regulatory networks, protein-protein interactions, and metabolite-pathway relationships, identifying hub transcription factors and key regulatory modules that coordinate drought tolerance responses [110]. Notably, simultaneous visualization of cross-omics relationships can be attained by multi-dimensional mapping of drought-relevant omics layers, which in most cases is achieved via machine learning-mediated integration pipelines and dimensionality reduction methods and facilitates the connection between genotype and phenotype in water-deficient environments [11]. Recent multi-omics single-cell studies continue to develop this paradigm with their production of high-resolution cellular maps in which clustering and trajectory analyses identify tissue specific cell-type drought responses [111]. Interactive dashboards and integrative visualization platforms have been applied in drought omics research to enable dynamic exploration of multi-omics datasets. For example, interactive multi-omics visualization platforms and curated plant stress databases integrate drought-responsive gene expression, protein interaction, and metabolite data across multiple crop species, enabling researchers to query stress-specific molecular signatures, compare responses across genotypes and stress conditions, and generate testable hypotheses without requiring advanced bioinformatics expertise [112]. These visualization structures aid systems level interpretation of drought stress by assembling heterogeneous information into coherent analysis schemes and thus speeding up the discovery of drought-sensitive genes, metabolic programs and adaptive systems that govern crop resistance.

## 4. Applications in Drought Stress Mitigation

### 4.1. Biomarker Discovery for Drought Tolerance

**Figure 3:**
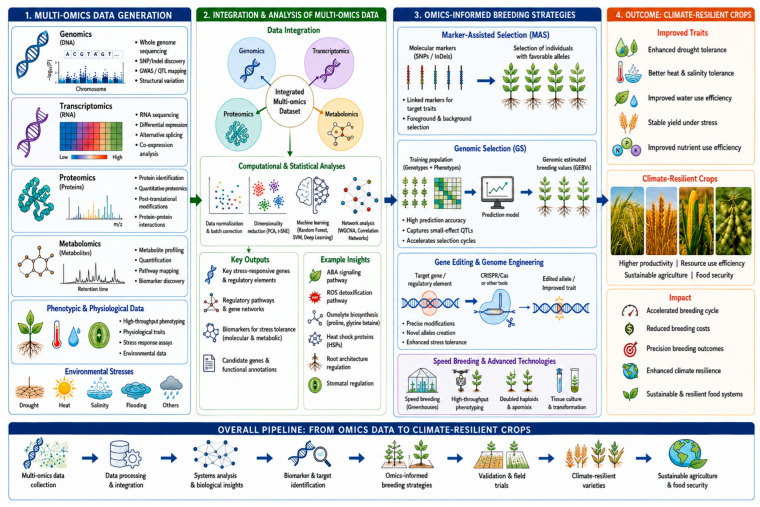
**Omics-informed strategies for accelerating the development of climate-resilient crops.** Integration of multi-omics data (genomics, transcriptomics, proteomics, and metabolomics) enables the identification of key genes, regulatory pathways, and biomarkers associated with stress tolerance. These insights support advanced breeding approaches, including marker-assisted selection, genomic selection, and gene editing, ultimately facilitating the development of crops with improved resilience to environmental stresses such as drought.

Biomarker discovery for drought tolerance has recently evolved from single gene screening to integrated omics frameworks because drought response is highly polygenic, dynamic and tissue-dependent involving concerted alterations in DNA variation levels, transcript abundance, protein accumulation, metabolite flux and regulatory network architecture (Figure 3). In this respect, a good drought biomarker is not simply a molecule that changes in response to water deficit, but a reproducible molecular signature that correlates with adaptive performance such as water-use efficiency, osmotic adjustment, membrane protection, antioxidant capacity, stomatal regulation, maintenance of photosynthesis and finally, yield stability under stress. Recent omics analysis showed that metadata and transcriptome analysis identified signs of drought response in conservatively selected genotypes including a core set of 3080 genes in rice. Some of the key candidates such as *LEA4-5*, *LEA14*, *OsRab16A*, *OsADF3*, *OsPP108* and *GBF3* were found to be potential candidates’ biomarkers based on being the central roles in the process of drought tolerance and ABA dependent stress [44]. In addition to transcript markers, metabolomics is also proving particularly robust in the case of biomarker prioritization (the metabolites are downstream of phenotype and tend to combine upstream genomic and environmental influences). A transcriptomic/metabolomic study reported on sugarcane presented the identification of 157 drought-responsive genes and 18 metabolites associated with them, co-expression analysis using these genes gave tryptophan, proline and 6-methylquinine (6-MQ) as metabolic hubs [113]. This is significant since proline and proline-related amino acid signatures, which have been long linked to osmoprotection, redox buffering, and stress recovery, can now be assessed through multi-omics technology and no longer be used as either a single readout. Similarly, the multi-omics analysis of rapeseed disclosed that cuticular wax biosynthesis, lipid metabolism and hormone signalling were linked to drought tolerance and *CER1*, *MYB*, *FAR3*, and *MAH1* were key genes [46]. In cotton, integrated transcriptome-metabolome analysis has been used to identify the genotype-specific drought response and the key transcription factor families (*HSF*, *bHLH*, *C2H2*, *B3*, *Tify*, *AUX/IAA*, *ARR-B*), which underlines the fact that drought tolerance is controlled by regulatory networks instead of single gene markers [47]. Another important advancement is the discovery of post-transcriptional regulation as a layer of biomarkers, a multi-omics approach to wild soybeans has linked drought adaptation to alternative splicing and has identified variations in isoforms of *FT1*, *CCR1L*, *RPL18* and their close neighbours as candidate markers [45]. This shows that drought tolerance is a factor that is not only dependent on the levels of expression of genes but also on the stress-specific transcript isoforms. Moreover, recent methodological efforts have highlighted that multi-omics integration and machine learning will tend to be the best predictive drought biomarkers due to the ability of integrative models to bridge the gap between genotype and field-relevant phenotypes. A study of wheat using RF-Boruta machine learning technique based on transcriptomics data showed how the selection of features can be used to refine large DEG molecules into a smaller group of key drought-responsive genes with increased predictive power [11].

### 4.2. Breeding Strategies Informed by Omics

Breeding strategies based on the knowledge and new approaches of omics have revolutionized the improvement of drought resilience from conventional phenotype-based selection to precision molecular breeding by providing marker-assistance selection (MAS) and genomic prediction frameworks that harness genome-wide and multi-layered molecular information, as depicted in Figure 3. MAS has been extensively applied to nonlinear pyramid drought-responsive quantitative trait loci (QTLs) and SNP markers characterized by genomics and multi-omics analyses that enable the efficient introgression of key genes controlling traits such as ABA signalling, osmotic adjustment and root architecture into elite cultivars and can accelerate the development of drought tolerant [114]. Importantly, the efficiency of MAS has been highly improved by omics-based marker discovery as the integration of genomics, transcriptomics and metabolomics-derived data sets allowed to identify robust biomarkers and regulatory networks related to stress tolerance which can be translated directly into selection markers for breeding programs [115]. In parallel, genomic prediction (genomic selection, GS) has become a powerful strategy to improve (complex) traits such as drought tolerance by estimating the genomic breeding value based on numerous molecular markers reaching early selection without being subject to extensive phenotyping. However, recent research has shown that prediction accuracy is markedly increased when multi-omics layers such as transcriptomics and metabolomics are included in genomic prediction that catch intermediate molecular phenotypes and intricate biological interactions underlying drought responses [116]. Similarly, in wheat-*Thinopyrum* introgression lines, multi-omics analysis combining ion transporter gene expression with seedling phenotyping has uncovered genotype-dependent salt tolerance mechanisms, demonstrating the value of integrated approaches for identifying precise markers in stress breeding [117].

Advanced computational approaches (machine learning and deep learning models), that further improve GS by incorporating the integration of heterogeneous datasets, modelling of non-linear genotype-phenotype relationships, will be able to provide more accurate predictions of drought-adaptive traits under the above-mentioned diverse environmental situations [11]. In this way, with such integrative pipelines, breeding can be transitioned to use genome-assisted breeding and further to AI powered breeding, where multi-omics-informing targets are tested, edited (via CRISPR), and introduced into breeding populations with more accuracy and less breeding cycles [118].

### 4.3. Microbiome Interactions Under Drought

Rhizosphere microbiome interactions represent an increasingly recognized dimension of plant drought adaptation, with metagenomic approaches providing high-resolution characterization of microbial community composition and functional potential under water deficit conditions. Shotgun metagenomic sequencing studies have demonstrated that drought stress selectively restructures the rhizosphere microbiome, enriching beneficial taxa including Streptomyces, Leifsonia, and members of the Rhizobiaceae, which contribute to drought adaptation through phytohormone production, osmolyte generation, and activation of stress-responsive pathways in host plants [119]. These community shifts are not random but are actively modulated by plant root exudates, which recruit specific microbial consortia capable of enhancing nutrient uptake, cellular homeostasis, and water-use efficiency under drought conditions [120]. Metagenomic functional profiling has further revealed enrichment of genes associated with nutrient cycling, stress resistance, and metabolic interactions within drought-restructured rhizosphere communities, establishing functional links between microbial community composition and plant performance under water deficit [121]. It is important to note, however, that most studies demonstrating beneficial effects of drought-associated microbiome shifts or microbial inoculations on plant performance have been conducted under controlled greenhouse or growth chamber conditions, and robust field-scale validation remains limited [122]. Under controlled conditions, inoculation with specific plant growth-promoting rhizobacteria (PGPR) strains has been shown to improve plant biomass, water-use efficiency, and stress tolerance through mechanisms including phytohormone production, nutrient solubilization, reduction of oxidative damage, and induction of systemic tolerance in host plants [123]. Drought-responsive fungal communities, or mycobiomes, have similarly been shown under controlled conditions to shift toward generalist taxa that promotes root growth and activate ABA signalling pathways, highlighting the multi-kingdom nature of microbially mediated drought adaptation. The plant-microbiome holobiont concept further frames drought adaptation as an integrated process involving host genetic predisposition, microbiome functional contributions, and resource-use efficiency [124].

Translating these promising findings to field-scale applications remains a significant challenge. Microbiome engineering strategies, including the design of synthetic microbial communities, targeted PGPR inoculation, and manipulation of root exudate chemistry to recruit beneficial microbiomes, are currently at early stages of development and have demonstrated variable efficacy under field conditions due to the complexity of soil environments, competition from indigenous microbial communities, and genotype-by-microbiome-by-environment interactions [122,125,126]. Future research should prioritize field-scale validation of microbiome-based drought mitigation strategies across diverse soil types, cropping systems, and climate zones before their adoption in commercial crop improvement programs.

### 4.4. Precision Agriculture and Climate-Smart Strategies

The integration of multi-omics data with sensor-based phenotyping and predictive models has progressively been established to enhance drought resilience and improve resource use in situations of water-limited conditions. The convergence of both high-throughput omics platforms (genomics, transcriptomics, proteomics, metabolomics) and high-resolution phenotyping systems of UAV-based remote imagery, hyperspectral sensors and ground-based Internet of Things (IoT) devices have provided the means to integrate the molecular responses to drought-induced effects with ecosystem [10] This combination allows the discovery of key drought-adaptive pathways including ABA signalling, ROS detoxification, and heat shock protein responses as well as allows dynamic measurement of genotype-by-environment interaction, which is essential to climate-smart crop enhancement [10]. Sensor platforms, like soil moisture sensors, environmental monitors, and plant stress, can be used to create the stream of continuous data, which, when combined with omics-discovered knowledge, can be used to characterize plant water-use efficiency and stress-levels in specific ways, thereby providing the opportunity to implement targeted interventions and minimize unnecessary resource investments [127]. Notably, the use of predictive models that involve machine learning and artificial intelligence has transformed irrigation scheduling and optimization of resources by examining multi-source information such as soil moisture, weather, crop physiological data and historical irrigation data to predict with high accuracy the water need and the level of stress when needed by the plants [128]. As an example, LSTM-based AI-driven decision-support systems that are fed with hyperspectral phenotyping measurements were shown to effectively detect the levels of drought stress and automatically regulate irrigation, leading to a higher water-use efficiency and crop resiliency [129]. Similarly integrated IoT based frameworks of sensor networks, remote sensing and machine learning algorithms support adaptive irrigation strategies adapting water application strategies on a real-time basis with a massive potential of reducing water consumption and yet sustaining or improving crop yield [130]. The inclusion of multi-omics information in such predictive systems also increases the robustness of the models by adding mechanistic information of stress-responsive pathways, thereby enabling more accurate genotype-to-phenotype predictions and helping the development of digital agriculture solutions that support climate resilient agriculture [118]. In addition, new tools like digital twins and crop modelling with data incorporate the environment, phenotypic, and molecular data to predict crop response to different drought conditions and, therefore, enable proactive decisions and effective resource distribution of precision-based agriculture [131].

## 5. Case Studies

### 5.1. Wheat: Multi-Omics Dissection of Drought Tolerance

**Figure 4:**
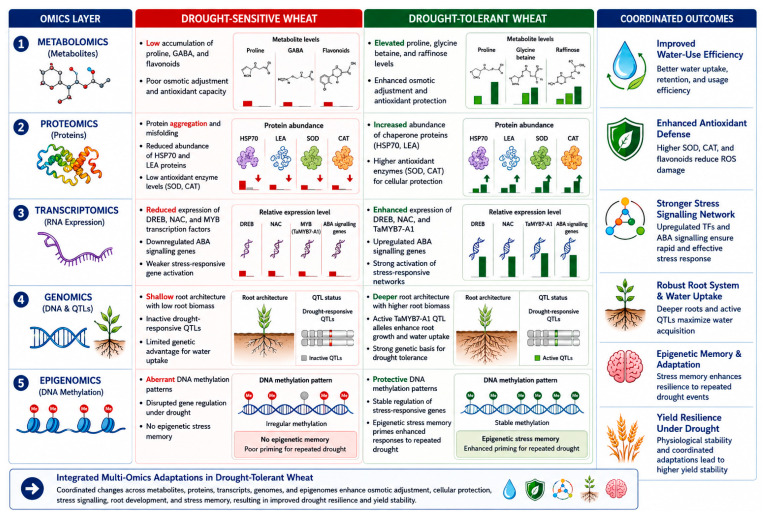
**Comparative multi-omics profile of drought-sensitive and drought-tolerant wheat across five omics layers.** Drought-sensitive wheat is characterized by low proline, GABA, and flavonoid accumulation (metabolomics), protein aggregation and reduced HSP70 and LEA protein abundance (proteomics), reduced expression of *DREB*, *NAC*, and *MYB* transcription factors and downregulated ABA signalling genes (transcriptomics), shallow root architecture with inactive drought-responsive QTLs (genomics), and aberrant DNA methylation patterns without epigenetic stress memory (epigenomics). In contrast, drought-tolerant wheat exhibits elevated proline, glycine betaine, and raffinose levels supporting osmotic adjustment, increased abundance of chaperone proteins (HSP70) and antioxidant enzymes (SOD, CAT) for cellular protection, enhanced *DREB*, *NAC*, and *TaMYB7-A1* expression for effective stress signalling, deeper root systems driven by active *TaMYB7-A1* QTL alleles improving water uptake, and protective DNA methylation patterns forming an epigenetic stress memory that primes enhanced responses to repeated drought. These coordinated multi-omics adaptations contribute to improved water-use efficiency, antioxidant defense, physiological stability, and yield resilience under drought conditions.

The recent multi-omics research on wheat has presented detailed information on drought tolerance through genomic, transcriptomic, proteomic, metabolomic and phenomic research, as shown in Figure 4. Genomic studies, especially the GWAS and high-density SNP studies, have revealed many QTLs related to root architecture and water-use efficiency with candidate genes like *TaMYB7-A1* that increase the depth of roots and drought resistance [26]. RNA-seq transcriptomic studies have shown that drought is reprogrammed with thousands of differentially expressed genes regulated in ABA signalling, osmotic adjustment and ROS detoxification pathways [132]. At the proteomic level, the LC-MS/MS data has shown accumulation of antioxidant enzymes i.e., superoxide dismutase and catalase and proteins related to stress that maintains cells stability in conditions of water deficit [133]. Metabolomic profiling confirmed elevated concentrations of key osmoprotectants consistent with osmotic adjustment and antioxidant defense responses described in Section 2.4, further validating these metabolites as drought biomarkers in wheat [134]. Moreover, UAV-based imaging and thermal sensors have also facilitated high-throughput phenomics that have allowed non-destructive measurements of canopy temperature, biomass and water-use efficiency, which confirm that drought-tolerant genotypes have cooler canopies and are physiologically more stable in a droughty environment [135]. Multi-omics combines molecular processes and phenotypic traits in wheat; it enhances precision breeding to produce wheat varieties that are drought resistant. A summary of these multi-omics results regarding drought tolerance in wheat is also provided (Table 2).

### 5.2. Rice: Genomic and Transcriptomic Insights into Water Stress

Understanding the genetic and molecular basis of drought tolerance in rice has been of top priority for research in the plant sciences with the advent of high-throughput multi-omics technologies. Recently, several studies have identified the importance of genomics and transcriptomics in drought tolerance in rice. *DRO1* significantly contributes to the development of deep rooting and enhanced water uptake through QTL-based mapping in rice [23]. Singh et al. [22] also described better haplotypes of *OsDREB1C* which confers drought tolerance and better grain-yield in the stressful environment. Another genomic study revealed the existence of numerous QTLs that were associated with water-use efficiency and osmotic adjustment in various rice germplasm [141]. Transcriptomic analyses have shown large-scale changes of gene expression in rice during drought. Tyagi, Kumar and Mohapatra [142] found thousands of differentially expressed genes that are involved in redox-homeostasis and ABA signalling during drought-stress responses. The meta-analysis found important genes and pathways that are related to drought tolerance in rice [143]. In another RNA-seq experiment, *LEA4-5*, *LEA14*, *OsRab16A*, *OsADF3*, *GBF3* and *OsPP108* genes were strongly induced in tolerant genotypes which increase their capacity to retain water and offer cellular protection [44]. Further details of genomics and transcriptomic studies are summarized (Table 3).

### 5.3. Maize: Metabolomic and Phenomic Contributions to Resilience

Metabolomics and phenomics have also played an important role in improving the knowledge of drought resilience in maize by integrating biochemical responses to the field level performance. Recent metabolomics analysis in maize identified drought-responsive metabolites including proline, tryptophan, and phenylalanine as regulators of ROS scavenging and hormone signalling pathways, extending the osmolyte accumulation patterns described in Section 2.4 to maize-specific biochemical responses under water deficit [61]. Rhizosphere metabolomics analysis demonstrated that drought suppresses soil metabolites and interaction of microbes, which leads to the significant decrease of biomass and manipulates plant adaptation mechanisms to stress [150]. Continuous monitoring of canopy traits, dynamism of maize growth stages and stress responses in high-throughput phenomics with UAV-based imaging enhanced the effectiveness of drought screening during maize breeding [84]. Field based phenomics trials revealed that drought tolerant maize lines are characterized by a superior canopy temperature regulation, photosynthesis and yield stability that facilitate phenotyping as an efficient method of selecting genotypes that are resilient [151]. Further detailed summary of key metabolomic and phenotypic traits associated with drought resilience in maize is presented in Table 4.

### 5.4. Legumes: Symbiotic Interactions Under Drought Conditions

Legumes have a special adaptive benefit during drought stress due to symbiotic interaction with rhizobia and arbuscular mycorrhizal fungi (AMF) that is important to increase nutrient uptake, stress resistance, and general plant wellbeing. In the ideal scenario, the symbiosis between legumes and rhizobia allows a process of biological nitrogen fixation (BNF), where atmospheric nitrogen is transformed into ammonia in root nodules which enhances the plant growth significantly and decreases the use of artificial fertilizers [155]. A recent study showed that root exudates, particularly flavonoids and other secondary metabolites, promote nitrogen fixation by modulating rhizosphere microbial community composition and enhancing rhizobial recruitment and function [156]. Another study has shown that legumes actively control symbiotic interactions during drought by complex physiological and molecular mechanisms. The pre and post infection control system can enable the host plants to select only the efficient rhizobial strains, which guarantee the best symbiotic working under stress [157] A study by He, Van Dingenen [158] has shown that a network of mycorrhizal can transfer host specific signalling molecules like flavonoids over long distances to enhance rhizobial recruitment and nodulation efficiency during stress. Similarly, Álvarez-Aragón, Palacios and Ramírez-Parra [159] also found that rhizobial symbiosis has a considerable effect in drought tolerance by increasing the nitrogen fixation, water-use efficiency, and antioxidant enzyme activity of *Vicia sativa* and *Pisum sativum*. These studies indicate that complex symbiotic interactions between chemical signalling, nutrient dynamics and microbial diversity are the drivers of drought tolerance in legumes. A summary of drought-responsive symbiotic mechanisms and is presented in Table 5. A comparative synthesis of conserved and species-specific drought-responsive mechanisms identified through multi-omics studies across wheat, rice, maize, and legumes is presented in Table 6.

Comparative analysis of multi-omics findings across wheat, rice, maize, and legumes reveals both broadly conserved and species-specific drought-responsive mechanisms. At the conserved level, ABA-mediated stomatal regulation, *DREB* and *NAC* transcription factor activation, LEA protein accumulation, proline-based osmotic adjustment, and antioxidant enzyme upregulation represent core drought tolerance mechanisms shared across all four crop groups, reflecting their fundamental roles in cellular protection and water deficit signalling. Similarly, epigenetic regulation through DNA methylation and miRNA-mediated gene silencing appears to be a conserved regulatory strategy across species, though the specific loci and small RNA species involved are largely species-specific [175]. At the species-specific level, legumes are uniquely distinguished by their capacity for symbiotic drought adaptation through rhizobial nitrogen fixation and arbuscular mycorrhizal fungal associations, mechanisms entirely absent in cereals [178]. Root system architecture adaptation through deep rooting QTLs is conserved in principle across all species but governed by distinct genomic loci in each crop, with *DRO1* in rice, *TaMYB7-A1*-associated root depth QTLs in wheat, and root cortical aerenchyma loci in maize representing parallel but genetically independent solutions to the same adaptive challenge [172]. These comparative insights highlight the value of cross-species multi-omics analyses for identifying transferable drought tolerance mechanisms and species-specific targets for precision breeding.

## 6. Challenges and Limitations

Although multi-omics approaches have substantially advanced our understanding of plant adaptation to drought stress, their routine application in crop discovery and breeding programs remain constrained by several interconnected technical, computational, biological, economic, and ethical challenges.

### 6.1. Technical and Data Integration Challenges

A fundamental technical challenge lies in the heterogeneity of data generated across omics platforms. Genomics, transcriptomics, proteomics, metabolomics, phenomics, and microbiome datasets differ markedly in sensitivity, resolution, dynamic range, and experimental design, making their integration inherently complex, particularly under variable field drought conditions where stress intensity and duration are difficult to standardize [179]. Each omics layer captures biological information at a different molecular scale and temporal resolution, and the absence of universally accepted data formats and metadata standards further complicates cross-platform integration. For example, transcriptomic data generated by RNA-seq may not directly correspond temporally with proteomic or metabolomic snapshots taken at different stress time points, introducing biological inconsistencies that confound integrated analyses. Standardization of experimental protocols, sampling strategies, and data reporting formats across omics disciplines is therefore urgently needed to improve data comparability and reproducibility.

### 6.2. Computational and Bioinformatics Challenges

The computational demands of processing, storing, and integrating large-scale multi-omics datasets represent a significant barrier, particularly for research groups in low- and middle-income countries with limited access to high-performance computing infrastructure. Multi-omics datasets are characteristically high-dimensional, often containing missing values, technical noise, and batch effects arising from differences in sequencing platforms, laboratory protocols, or sample processing conditions [179,180]. Addressing these issues requires sophisticated bioinformatics pipelines incorporating robust normalization methods such as quantile normalization, variance stabilization, and empirical Bayes correction, as well as dimensionality reduction approaches including PCA and PLSR. However, many of these tools require specialized bioinformatics expertise that is not consistently available across breeding programs. Furthermore, the risk of model overfitting in machine learning-based integration approaches is substantial when training datasets are small relative to the number of molecular features, necessitating careful cross-validation, feature selection, and independent replication to ensure biological validity of predictive models.

### 6.3. Biological Complexity and Transferability Challenges

The biological complexity of drought tolerance itself presents a major challenge for multi-omics interpretation. Drought tolerance is a highly polygenic, dynamic, and context-dependent trait governed by intricate non-linear interactions among genes, proteins, metabolites, and environmental factors. Genotype × environment × management interactions, feedback regulatory loops, epigenetic stress memory, and pathway crosstalk among ABA signalling, ROS detoxification, osmotic adjustment, and growth regulatory networks can result in the same drought-tolerant phenotype arising from distinct molecular states in different genotypes or environments [179]. This phenomenon, known as phenotypic convergence through divergent molecular mechanisms, severely complicates biomarker discovery and reduces the transferability of molecular markers and predictive models across species, tissues, developmental stages, and field environments. Single-cell and spatial omics approaches, while promising, are still in early stages of application in crops and have not yet fully resolved the challenge of tissue-specific and cell-type-specific heterogeneity in drought responses.

### 6.4. Economic and Accessibility Challenges

Large-scale multi-omics integration requires access to high-throughput sequencing platforms, high-resolution mass spectrometry for proteomics and metabolomics, advanced phenotyping infrastructure including UAV-based imaging and hyperspectral sensors, and cloud or high-performance computing facilities. The combined cost of these technologies remains prohibitive for many public breeding programs and agricultural research institutions, particularly in drought-affected regions of sub-Saharan Africa and South Asia where the need for drought-resilient crop varieties is most acute. The requirement for interdisciplinary expertise spanning molecular biology, bioinformatics, statistics, agronomy, and data science further limits the capacity of many institutions to implement integrated omics pipelines. Investment in shared infrastructure, open-access databases, and capacity building programs is therefore essential to democratize access to multi-omics technologies in crop improvement [103].

### 6.5. Ethical, Regulatory, and Data Governance Challenges

The large-scale generation and sharing of genomic and multi-omics data from crop genetic resources raise important ethical and regulatory questions concerning data ownership, intellectual property rights, benefit sharing, and biosafety. Tensions between open science principles and commercial interests can restrict data sharing and collaborative research, limiting the collective advancement of drought-resilient crop development. Regulatory frameworks governing the deployment of gene-edited crops developed through CRISPR-based omics-informed breeding vary substantially across countries, creating additional barriers to the translation of omics discoveries into commercially available drought-tolerant varieties [181]. Establishing transparent, equitable, and internationally harmonized data governance frameworks and regulatory pathways will be essential to facilitate responsible and inclusive use of multi-omics technologies in global agricultural systems [179].

## 7. Future Perspectives

Future improvements in drought-stress research will increasingly rely on new generation omics technologies, specifically single cells and spatial omics, which allow investigating cell-type and tissue-specific responses commonly masked in bulk analyses. Recent studies have shown that single-cell transcriptomics can be used to understand the unique cellular states in drought stress and recovery whereas spatial transcriptomics in crops like rice identified localized regulatory networks and key genes (e.g., *HMGB1*) linked to root adaptation under water limited conditions and therefore may be used to identify the exact targets of interest [111]. Building on current machine learning frameworks, next-generation AI-driven predictive models will increasingly incorporate multi-modal data streams including environmental, phenotypic, and molecular inputs to reveal complex regulatory trends and enable accurate trait prediction across diverse field environments. In addition, by combining multi-omics with the use of digital agriculture technologies, such as Internet of Things (IoT) sensors, smart irrigation systems, and drone-based phenotyping, it will be possible to monitor the physiological conditions of plants, soil moisture, UAV-based sensor networks and imaging have already proven to be highly efficient in the early detection of droughts and massive phenotyping [127]. In the future, towards 2030, convergence of these technologies with genome editing, systems biology and predictive breeding strategies is required to develop climate-resilient crops offering breeders the ability to design crops that retain productivity under changing water conditions. However, achieving this vision, better standardization, data interoperability, cost effective infrastructure, and translation of the information garnered by omics into workable breeding pipelines will be needed so that the understanding of drought can be scaled and applied across different agricultural systems [182].

## 8. Conclusions

Multi-omics technologies have fundamentally transformed our understanding of drought tolerance in crops, shifting the research paradigm from single-gene approaches to integrated system-level analyses that capture the full complexity of molecular stress adaptation. Genomics, transcriptomics, proteomics, metabolomics, epigenomics, phenomics, and microbiome analyses collectively reveal that drought tolerance is governed by highly orchestrated interactions among gene regulatory networks, protein dynamics, metabolic reprogramming, and physiological adaptations rather than sequential linear pathways. Conserved mechanisms including ABA-mediated stomatal regulation, *DREB* and *NAC* transcription factor activation, LEA protein accumulation, proline-based osmotic adjustment, antioxidant enzyme upregulation, and epigenetic stress memory operate across major crop species, while species-specific adaptations such as legume symbiotic nitrogen fixation and crop-specific deep rooting QTLs provide additional layers of targetable drought resilience. Strategically, translating these discoveries into breeding outcomes requires coordinated integration of marker-assisted selection, genomic selection, and CRISPR-based gene editing with omics-informed target identification, supported by machine learning frameworks that improve selection accuracy and enable genotype-to-phenotype predictions across diverse environments. The convergence of single-cell and spatial omics, AI-driven predictive modelling, digital agriculture platforms, and IoT-based monitoring systems holds transformative potential for designing crops resilient to future climate scenarios. Realizing this potential requires standardization of omics protocols, open-access databases, investment in shared infrastructure and capacity building in low- and middle-income countries, and internationally harmonized data governance frameworks. Multi-omics thus represents not only a powerful research tool but a strategic cornerstone for advancing climate-smart agriculture and global food security in the face of accelerating climate variability.

## Figures and Tables

**Table 2:** Recent multi-dimensional omics tools and their applications for drought tolerance in wheat.

Omics-Field	Platform/Technology	Applications	References
Genomics	GWAS with 90K-SNP array	Identify 53 SNPs and 44 drought tolerance genes	[25]
	GWAS/QTL mapping	Determines SNP markers and genes of drought tolerance	[136]
Proteomics	LC–MS/MS	Determines drought-responsive proteins	[133]
Transcriptomic	MeRIP-seq	Determines the site of m6A-methylation and the drought-tolerant genes	[137]
	RNA-seq	Discovers differentially expressed drought-responsive genes	[138]
Metabolomics	GC–MS/LC–MS	Determines drought-responsive metabolites	[134]
Phenomics	UAV-based phenotyping	Identifies resistant varieties to drought	[135]
Epigenomics	MSAP analysis	Research DNA-methylation in adaptation to drought stress	[139]
Integrated omics	Multi-omics-tool	Determines the drought-responsive pathways, notable genes and metabolites	[140]

**Table 3:** Genomics and transcriptomic tools and their applications for drought tolerance in rice.

Omics-Field	Platform/Technology	Application	References
Genomics	SNP genotyping based on WGS/GWAS	Discovers SNPs marker of drought resistance	[144]
	GWAS and QTL mapping	Determines SNP markers and QTL of drought tolerance	[145]
	WGS and population-genomics analysis	Determines genomic-signatures and candidate genes	[146]
Transcriptomic	RNA-seq	Discovers differentially expressed genes	[147]
	Spatial transcriptomics of root	Discover tissue specific drought targets	[148]
	RNA-seq-mediated transcription-factor analysis	Identification and validation of drought-responsive genes	[149]

**Table 4:** Metabolomic and phenomic tools and their applications for drought tolerance in maize.

Omics-Field	Platform/Technology	Application	References
Metabolomics	LC–MS/GC–MS-based profiling	Determines root-metabolites sensitive to drought	[152]
	UPLC-MS/MS and transcriptomic analysis	Detection of 460 differentially accumulated metabolites	[153]
	Pathway level metabolic analysis	Identification of metabolic pathways for drought-resistance	[24]
Phenomics	Optical phenotyping with GWAS	Determines genetic-loci and drought-tolerance	[154]

**Table 5:** drought-responsive symbiotic mechanisms and its effects on legumes.

Symbiotic Component	Mechanism Under Drought	References
Rhizobia	Decreased nitrogenase activity and nodulation in drought	[155]
Rhizobial-symbiosis enhancement	Enhanced nitrogen-fixation and water use efficiency	[159]
Root exudates	Chemical signalling enhances rhizobial-recruitment in drought	[160]
Rhizobia and arbuscular mycorrhizal fungi	improve nutrient-uptake, root-growth and stress-responsive processes	[161]
Rhizobia and soil-microbiota	Alteration in microbial-communities	[162]
Rhizosphere-microbial symbiosis	Recruitment of specific microbes and improving root-function and nutrient uptake for drought tolerance	[162]

**Table 6:** Comparative synthesis of conserved and species-specific drought-responsive mechanisms identified through multi-omics approaches in major crop species.

Drought Mechanism	Pathway/Gene	Wheat	Rice	Maize	Legumes	Conservation Status
ABA-mediated stomatal closure	*PYR/PYL/RCAR-PP2C-SnRK2* cascade	✓	✓	✓	✓	Conserved across all species
Transcription factor activation	*DREB/CBF* family	✓ [132]	✓ [22]	✓ [119]	✓ [163]	Conserved across all species
Transcription factor activation	*NAC* family	✓ [43]	✓ [44]	✓ [38]	✓ [164]	Conserved across all species
Transcription factor activation	*MYB* family	✓ [26]	✓ [165]	✓ [38]	✓ [163]	Conserved across all species
Cellular protection	LEA protein accumulation	✓ [133]	✓ [44]	✓ [166]	✓ [167]	Conserved across all species
Osmotic adjustment	Proline accumulation	✓ [134]	✓ [60]	✓ [61]	✓ [159]	Conserved across all species
Osmotic adjustment	Glycine betaine accumulation	✓ [134]	✓ [60]	✓ [61]	✓ [168]	Conserved across all species
Antioxidant defense	SOD, CAT, peroxidase upregulation	✓ [133]	✓ [142]	✓ [169]	✓ [159]	Conserved across all species
Root system adaptation	Deep rooting QTLs	✓ [18]	✓ [23]	✓ [170]	✓ [171]	Conserved; species-specific QTL loci
Water-use efficiency	Stomatal conductance regulation	✓ [83]	✓ [141]	✓ [88]	✓ [172]	Conserved across all species
Epigenetic regulation	DNA methylation changes	✓ [173]	✓ [68]	✓ [76]	✓ [174]	Conserved; context-dependent
Post-transcriptional regulation	miRNA-mediated gene silencing	✓ [78]	✓ [78]	✓ [76]	✓ [175]	Conserved across all species
Hormone signalling	ABA biosynthesis via NCED	✓ [176]	✓ [176]	✓ [176]	✓ [176]	Conserved across all species
Symbiotic adaptation	Rhizobial nitrogen fixation	✗	✗	✗	✓ [159]	Legume-specific
Symbiotic adaptation	AMF-mediated nutrient uptake	✗	✗	✗	✓ [158]	Legume-specific
Cell wall remodelling	Lignin biosynthesis induction	✓ [57]	✓ [57]	✓ [57]	✓ [57]	Conserved; degree varies by species
Metabolic reprogramming	GABA accumulation	✓ [62]	✓ [62]	✓ [62]	✓ [62]	Conserved across all species
Genome editing target	CRISPR-edited stress regulators	✓ [177]	✓ [29]	✓ [31]	✓ [40]	Species-specific gene targets

✓ = documented in this species; ✗ = not applicable or not documented.

## Data Availability

No new data was created or analysed in this study. Data sharing is not applicable to this article.
